# Comparative analysis of PI3K-AKT and MEK-ERK1/2 signaling-driven molecular changes in granulosa cells

**DOI:** 10.1530/REP-24-0317

**Published:** 2025-01-21

**Authors:** Vijay Simha Baddela, Marten Michaelis, Xuelian Tao, Dirk Koczan, Julia Brenmoehl, Jens Vanselow

**Affiliations:** ^1^ Research Institute for Farm Animal Biology (FBN), Dummerstorf, Germany; ^2^ Institute of Immunology, University of Rostock, Rostock, Germany

**Keywords:** ovary, granulosa cells, PI3K-AKT, MEK-ERK1/2, sex steroids, metabolism

## Abstract

**In brief:**

PI3K-AKT signaling activates steroidogenesis by inducing estradiol and progesterone production, while MEK-ERK1/2 signaling regulates steroidogenesis by inhibiting estradiol and inducing progesterone production in granulosa cells (GCs). Both pathways are essential for glycolytic and mitochondrial metabolism in these cells.

**Abstract:**

The PI3K-AKT and MEK-ERK1/2 signaling pathways are integral to fundamental cellular processes, such as proliferation, viability and differentiation. In GCs, these pathways are activated by follicle-stimulating hormone (FSH) and IGF1 through respective receptors. We investigated the comparative transcriptome changes induced by the AKT and ERK (ERK1/2) pathways using corresponding inhibitors in GCs. GCs isolated from antral follicles showed positive signals for phospho-AKT and phospho-ERK proteins. Treatment of cultured GCs with FSH and IGF1 induced phospho-AKT and phospho-ERK levels. Transcriptome analysis revealed 1436 genes regulated by AKT and 654 genes regulated by the ERK pathway. Among these, 94 genes were commonly downregulated and 11 genes were commonly upregulated in both datasets, while 110 genes were oppositely regulated. Bioinformatics analysis revealed that the inhibition of the PI3K-AKT and MEK-ERK pathways downregulates key reproductive processes and upstream molecules. Notably, AKT inhibition affected FSH, ESRRG and HIF1 pathways, while ERK inhibition impacted CG, FOS, TGFβ, EGR1 and LH pathways. Transcriptome data showed that genes related to estradiol production were inhibited by ERK and induced by the AKT pathway. This was verified by radioimmunoassays, and mRNA and protein analysis of *CYP19A1* and *STAR* genes. In addition, transcriptome data suggested the downregulation of glucose metabolism in GCs. Using validation experiments, we confirm that both pathways are essential for glucose uptake, lactate production and mitochondrial activity in GCs. These data provide a resource for informing future research for analyzing various novel candidate genes regulated by the AKT and ERK pathways in GCs and other cell types.

## Introduction

**Figure 1:**
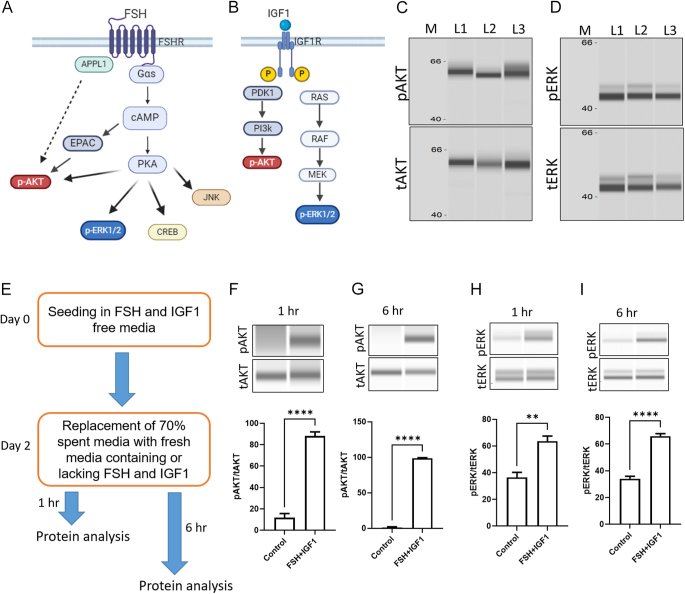
Status of AKT and ERK signaling pathways in granulosa cells of freshly isolated (ovarian follicular) and cultured granulosa cells. (A) and (B) Graphical representation of AKT and ERK signaling pathways activated by FSH and IGF1 in granulosa cells. These graphics were generated using bioRender.com. (C) and (D) Phospho- and total protein signals of AKT and ERK proteins, respectively, in freshly isolated granulosa cells derived from 2 to 6 mm ovarian follicles. M lane indicates molecular size. Lanes L1–L3 indicate three different replicates. (E) Schematic representation and timeline of granulosa cell culture used for the phosphoprotein analysis shown in (F), (G), (H) and (I). (F) and (G) Quantification of the phospho- and total AKT proteins in granulosa cells cultured in the absence or presence of FSH and IGF1 at 1 and 6 h time points, respectively. (H) and (I) Detection and quantification of the phospho- and total ERK proteins in granulosa cells cultured in the absence or presence of FSH and IGF1 at 1 and 6 h time points, respectively. Data are presented in mean ± SEM values (*n* = 5) and analyzed by unpaired *t*-test. Probability values <0.05 were considered statistically significant and were designated with asterisk symbols to inform the strength of significant difference (**P < 0.01; ****P < 0.0001). The *n* value indicates the number of independent cell culture experiments analyzed using granulosa cells collected on different days.

Ovarian follicles are among the most prolific tissues, with rapid cellular proliferation and differentiation to enable the growth and maturation of oocytes for fertilization. Signaling of FSH (follicle-stimulating hormone) and IGF1 (insulin-like growth factor 1) through respective receptors in ovarian granulosa cells is crucial for the development of dominant follicles in various species, including humans and cows. Synergic actions of FSH and IGF1 induce proliferation and estradiol production in granulosa cells (Mani *et al.* 2010, Zhou *et al.* 2013, Baumgarten *et al.* 2014). Knocking out *FSH* or *IGF1* genes or their receptors leads to infertility (Kumar *et al.* 1997, Abel *et al.* 2000, Baumgarten *et al.* 2017). FSH receptors typically activate G proteins by splitting the βγ dimer from the α subunit (Casarini & Crépieux 2019). Gαs proteins activate adenylyl cyclase and elevate the cytoplasmic cAMP levels, which in turn act as an inducer for PKA (protein kinase A) and EPAC (exchange proteins directly activated by cAMP) signaling in rat granulosa cells (Wayne *et al.* 2007). It has been shown that FSH could also activate the AKT and ERK (ERK1/2) signaling pathways using intermediary molecules, which are mechanistically studied in human and rat granulosa cells (Casarini & Crépieux 2019, Thomas *et al.* 2011, Ulloa-Aguirre *et al.* 2018, Paradiso *et al.* 2021) (Fig. 1A). On the other hand, insulin and IGF1 activate AKT through the PI3K pathway and ERK signaling through the MAP kinase pathway (Hakuno & Takahashi 2018) (Fig. 1B). Studies have shown that AKT and ERK phosphorylation levels are significantly higher in granulosa cells of rats treated with both FSH and IGF1 than those treated with FSH or IGF1 alone (Zhou *et al.* 2013). A similar observation was made on the AKT phosphorylation in bovine granulosa cells, where FSH+IGF1 treatment did not further induce ERK phosphorylation compared to FSH or IGF1 alone (Mani *et al.* 2010), while it did in comparison with IGF1 treatment in another study (Ryan *et al.* 2008).

Activation of AKT signaling is essential for fertility as AKT1^−/−^ female mice showed impaired follicular development and oocyte growth. Granulosa cell expression of KITLG (KIT-ligand) and BCL2L1 (Bcl-2-like protein 1), which are involved in the proliferation and survival pathways, is significantly reduced in AKT-null mice (Brown *et al.* 2010). It has been shown that the PI3K-AKT pathway plays an essential role in the bidirectional communication between the cumulus and oocyte by mediating the phosphorylation of connexin 43, a gap junction protein (Shimada & Terada 2001, Ilha *et al.* 2015). In mural granulosa cells, PI3K-AKT signaling was found to induce numerous genes that mark cell survival, proliferation and steroidogenesis (Goto *et al.* 2009, Mani *et al.* 2010, Liu *et al.* 2021).

ERK signaling appears to be essential during the final stages of follicle development, where luteinizing hormone (LH) induces ovulation and corpus luteum formation. Granulosa-specific ERK1/2-null mice showed normal follicular development until the preovulatory follicular stage, where they were permanently arrested and did not respond to superovulation treatment (Fan *et al.* 2009). Oocytes in these mice did not show cumulus expansion. Similar observations were made in mice treated with the ERK chemical inhibitor PD0325901, which inhibits ERK phosphorylation by an upstream MEK protein (Siddappa *et al.* 2015). In a previous study, we reported that ERK signaling reduces *FOXL2* (forkhead box protein L2) and *CYP19A1* (aromatase) expression, leading to decreased estradiol production while simultaneously inducing *STAR* (steroidogenic acute regulatory protein) expression and progesterone production in cultured bovine granulosa cells (Baddela *et al.* 2023). In addition, we reported genome-wide gene expression changes induced by ERK signaling, identifying 654 gene clusters as significantly regulated, with an FDR-adjusted *P*-value of <0.05 and a fold change of |1.4|, upon ERK pathway inhibition (GSE225283). Bioinformatic functional annotation suggested that inhibition of ERK signaling could induce FSH signaling and inhibit LH signaling in granulosa cells, which was corroborated with the earlier estradiol and progesterone data.

While the regulation of the MEK-ERK and PI3K-AKT pathways in response to gonadotropins and growth factors has been well studied in granulosa cells, a comparative analysis of the transcriptomic changes induced by these two pathways has not been previously explored. In this study, we analyzed the transcriptomic effects of PI3K-AKT pathway inhibition and compared the significantly regulated genes with the previously published ERK-regulated transcriptome to identify uniquely or commonly regulated genes by these two signaling pathways. While transcriptome analyses of PD98059- and LY294002-treated cells were performed in separate experiments, each transcriptome experiment was included with its own experimental control. Therefore, the impact of the experimental variables, such as batch of reagents and genetics of cells, may not affect the functional interpretations of these pathways.

## Methods

### Collection of ovaries, isolation of granulosa cells and *in vitro* culture

The ovaries were obtained from a local abattoir (DANISH CROWN Teterower Fleisch GmbH, Germany) and transported to the laboratory in a phosphate-buffered saline (PBS) solution containing penicillin (100 IU/mL), streptomycin (100 μg/mL) and 0.5 μg/μL amphotericin. Granulosa cells were manually aspirated from 2 to 6 mm sized ovarian follicles using a 3-mL syringe and 18G needles. Only follicles with a transparent appearance and clear follicular fluid without debris were considered for granulosa cell aspiration. Viable cells were counted via the trypan blue exclusion method, and cells were cryopreserved in a freezing medium made of fetal calf serum containing 10% DMSO (Baufeld & Vanselow 2018).

On the day of culture, cryopreserved vials were thawed in a water bath at 37 °C and cells were washed using αMEM by centrifugation at 500 *g* for 3 min. The cell pellet was dissolved in αMEM containing 2 mM glutamine, 10 mM sodium bicarbonate, 20 mM HEPES, 4 ng/mL sodium selenite, 0.1% w/v BSA, 5 μg/mL transferrin, 10 ng/mL insulin, 1 mM non-essential amino acids, 100 IU/mL penicillin and 0.1 mg/mL streptomycin. Androstenedione (2 mM) was added to the media on the day of culture. In addition, FSH (20 ng/mL) and/or R3 IGF1 (50 ng/mL) was added either at the time of seeding or on day 2, depending on the experiment.

The cells were seeded at a density of 5 × 10^4^ cells per well in 48-well culture dishes, pre-coated with 0.02% collagen R (Serva, Germany) to promote healthy attachment. To determine the phosphorylation of AKT and ERK proteins, cells were seeded in growth media lacking FSH and IGF1 and cultured for two days. On day 2, 70% of the spent media was replaced with fresh media containing or lacking FSH and IGF1 and harvested after one or six hours. In contrast, cells were seeded in FSH- and IGF1-supplemented media to analyze the effect of inhibitors. On day 2, cells were treated with 50 μM of AKT inhibitor (LY294002), 50 μM of an ERK inhibitor (PD98059) or a combination of both by replacing 70% spent media with fresh media supplemented with inhibitors but lacking FSH and IGF1. After 2 h, 70% of the media was again replaced with fresh media containing FSH and IGF1, and cells were analyzed after 30 min or on day 4. DMSO was used as vehicle control in all the experiments that involved PD98059 and LY294002. Identical cell culture conditions were employed in a previous study, where the transcriptome analysis of PD98059-treated cells was performed (Baddela *et al.* 2023).

### Transcriptome analysis

The Bovine Gene 1.0 ST Arrays (Affymetrix, USA) were used for the transcriptome analysis. The RNA from DMSO- and LY294002-treated cells was isolated using the RNeasy Mini Kit (Qiagen, Germany). RNA quality was calculated using the Bioanalyzer Instrument (Agilent Technologies, USA), which revealed the RIN values ranging from 9.8 to 10.0 and RNA concentrations between 37 and 50 ng/μL, indicating negligible to no degradation of isolated RNA in all samples. Amplification and labeling steps were carried out using ‘GeneChip 3′ Amplification One-Cycle Target Labeling and Control Reagents’ (Affymetrix) following the manufacturer’s protocol. Hybridization was performed overnight in the GeneChip Hybridization Oven (Affymetrix), and the results were visualized using the Affymetrix GeneChip Scanner 3000. Subsequent analysis was performed using Transcriptome Analysis Console 4.0 (TAC4.0, Affymetrix, https://www.thermofisher.com) to identify differentially expressed genes. Analysis of variance (ANOVA) was used to calculate the *P*-value, which was additionally corrected by the FDR (false discovery rate, Benjamini–Hochberg) method integrated into TAC 4.0. A fold change threshold of |1.4|, coupled with an FDR < 0.05, was used as the cutoff to identify differentially expressed genes in the LY294002 vs control dataset. This selection criterion aligns with the cutoff parameters applied in our previously published PD98059 vs control dataset (Baddela *et al.* 2023), ensuring consistency across analyses. Ingenuity Pathway Analysis (IPA) was used to derive the enriched cellular functions and upstream molecules from the transcriptome data.

### RNA, cDNA and gene expression analysis

Total RNA was isolated from the cultured cells using the innuPREP RNA Mini Kit (Analytik Jena, Germany) following the manufacturer’s guidelines. The RNA concentration was measured with the help of a NanoDrop 1000 Spectrophotometer (Thermo Scientific, Germany), and cDNA was prepared using the SensiFAST cDNA Synthesis Kit (Bioline, Germany). qPCR analysis was performed using the SensiFAST SYBR No-ROX Kit (Bioline) and gene-specific primers listed in Table 1. The genes of interest were amplified in duplicates in a total volume of 12 μL using a LightCycler 96 Instrument (Roche, Germany). External standards for each gene of interest were generated using pGEM T-Vector cloning and verified by sequencing. Five different dilutions of the verified external standard (5 × 10^−12^ to 5 × 10^−16^ g DNA/reaction) were freshly prepared and co-amplified with each run. Melting point analysis was done after each run to check for amplification of the correct products. In addition, PCR products were verified by 3% agarose gel electrophoresis.

**Table 1:** qPCR primers.

Gene	Sequence		Size (bp)	NCBI accession no.
	Forward	Reverse		
*CYP19A1*	GCT​TTT​GGA​AGT​GCT​GAA​CCC​AAG​G	GGG​CCC​AAT​TCC​CAG​AAA​GTA​GCT​G	172	NM_174305
*STAR*	TTG​TGA​GCG​TAC​GCT​GTA​CCA​AG	CTG​CGA​GAG​GAC​CTG​GTT​GAT​G	236	NM_174189.2
*HSD3B*	TGT​TGG​TGG​AGG​AGA​AGG​ATC​TG	GCA​TTC​CTG​ACG​TCA​ATG​ACA​GAG	208	NM_174343
*FOXL2*	AGC​CAA​GTT​CCC​GTT​CTA​CG	GGT​CCA​GCG​TCC​AGT​AGT​TG	140	NM_001031750.1
*FSHR*	TCA​CCA​AGC​TTC​GAG​TCA​TCC​CAA​A	TCT​GGA​AGG​CAT​CAG​GGT​CGA​TGT​A	189	NM_174061
*RPLP0*	TGG​TTA​CCC​AAC​CGT​CGC​ATC​TGT​A	CAC​AAA​GGC​AGA​TGG​ATC​AGC​CAA​G	142	NM_001012682

### Western analysis

Protein quantification was performed using ProteinSimple’s WES instrument (Bio-Techne, USA) following the manufacturer’s guidelines. Cells were cultured in 48-well plates, washed using 1× PBS three times and lysed using 1× MPER lysis buffer (Thermo Fisher, USA). The lysate was centrifuged at 4 °C at 12,000 *g* for 3 min to collect the protein supernatant. Protein concentrations were determined using the Micro BCA protein quantification method (Thermo Fisher). Solutions such as wash buffers, blocking reagents, primary and secondary antibodies, and chemiluminescent substrates were distributed into predefined wells of the assay plate. The assay plates were then loaded onto the WES instrument for protein separation using a 12–230 kDa capillary separation module (SM-W001). The protein bands were detected using the software’s hydrodynamic range exposure feature. The primary antibodies used for the analysis are listed in Table 2. In addition, the anti-mouse (DM-002) and anti-rabbit (DM-001) secondary antibody modules were purchased from ProteinSimple, USA.

**Table 2:** Antibodies.

Antibody	Product no.	Company	Source
Aromatase	SM2222P	Acris (Origene), USA	Mouse
STAR	PA5-21687	Invitrogen, USA	Rabbit
Phospho-ERK1/2	9101S	Cell Signaling, USA	Rabbit
Total ERK1/2	4695S	Cell Signaling, USA	Rabbit
Phospho-Akt (Ser473)	9271S	Cell Signaling, USA	Rabbit
Total Akt	9272S	Cell Signaling, USA	Rabbit
Beta actin	SC47778	Santa Cruz, USA	Mouse

### Radioimmunoassays

Estradiol concentration in the conditioned media was determined using a modified competitive 3H-RIA with the tracer [2,4,6,7-3H] estradiol-17β, purchased from GE Healthcare (Germany). The intra- and interassay coefficients of variation were 6.9 and 9.9%, respectively, and the lower detection limit was 3 pg/mL. For analysis, 10 μL undiluted media were used. Progesterone concentrations were measured using an optimized direct competitive 3H-radioimmunoassay (RIA). The tracer [1,2,6,7-3H(N)] progesterone was procured from PerkinElmer (USA), and the rabbit-raised antibody was purified by chromatography. Radioactivity measurement was conducted in a liquid scintillation counter with an integrated RIA-calculation program (TriCarb 2900 TR, PerkinElmer), and the intra- and interassay coefficients of variation were 7.6 and 9.8%, respectively. The lower detection limit was 7 pg/mL.

### Glucose and lactate analysis

Glucose and lactate concentrations were measured in the cell-free spent culture media using an automatic enzymatic analyzer (ABX Pentra 400, HORIBA Medical, France) and the respective commercial kits. For glucose, Kit No. A11A01667, Axon Lab. AG, Germany, was used. For lactate, Kit No. A11A01721, Axon Lab. AG, was used. The interassay coefficients of variation for glucose and lactate were 0.9 and 0.6%, respectively.

### Mitochondrial membrane potential (MMP) analysis

MitoTracker staining of cells was performed following a previously described protocol (Tao *et al.* 2024). For JC-1 analysis, cultured cells were detached by incubation with 200 μL of ready-to-use Accutase solution (A6964, Sigma-Aldrich, USA) for 20 min at 37 °C. Cells were transferred into 1.5-mL tubes, washed twice with DMEM and incubated with JC-1 dye (T3168, Thermo Fisher Scientific) at a final concentration of 2 μM in DMEM for 30 min at 37 °C. Cells were washed three times in DMEM and analyzed in a flow cytometer with an excitation wavelength of 485 ± 10 nm (for both JC1 aggregates (red) and monomers (green)) and emission wavelengths at 590 ± 10 nm (for aggregates only) and 510 ± 10 nm (for monomers only). The fluorescence intensities were analyzed using the Kaluza 1.2 software (www.beckman.de). Forward versus side scattering gating was employed to analyze cell morphology and omit cell debris from the analysis, whereas fluorescence signals from propidium iodide staining were used to exclude the dead cells from the analysis.

### Statistical analysis

Statistical analyses and data visualization were performed using the GraphPad Prism 10.3 licensed software (www.graphpad.com). Cell culture experiments were performed in three to six biological replicates, as indicated in figure legends, using cells aspirated from ovaries collected on different days. All cell culture experiments contain two or three culture replicates at each condition, and the average of culture replicates was used for data analysis. Protein measurements involve the analysis of pooled lysates from culture replicates. Unpaired two-way *t*-tests were performed to compare the control and FSH+IGF1 treatments. The remaining data were analyzed using one-way ANOVA. Pairwise multiple comparisons were executed using Tukey’s post hoc tests. Data were presented as either the mean with SEM values or box plots. Probability values <0.05 were considered statistically significant and are designated with up to four asterisk symbols to inform the strength of the significant difference (*P < 0.05; **P < 0.01; ***P < 0.001; ****P < 0.0001).

## Results

### Induction and inhibition of PI3K-AKT and MEK-ERK1/2 signaling in cultured granulosa cells

FSH and IGF1 stimulate the AKT and ERK signaling pathways in ovarian granulosa cells, as indicated in Fig. 1A and B (Gonzalez-Robayna *et al.* 2000, Shiota *et al.* 2003, Ryan *et al.* 2008, Mani *et al.* 2010, Zhou *et al.* 2013). Western analysis of granulosa cells derived from antral follicles (2–6 mm), which are naturally exposed to FSH and IGF1 during follicular development, showed a positive signal for phospho-AKT and ERK proteins (Fig. 1C and D). To verify the phosphorylation of AKT and ERK under the present *in vitro* conditions, granulosa cells were seeded and cultured in IGF1 and FSH-free media for an initial two days. On day two, the media were replaced with fresh media containing 50 ng/mL of IGF1 and 20 ng/mL of FSH. The cells were lysed at 1 and 6 h time points and analyzed for AKT and ERK phosphorylation (Fig. 1E). Results showed that the FSH and IGF1 treatment significantly induced the AKT and ERK phosphorylation at both 1 and 6 h compared to control cells (Fig. 1F, G, H, I).

### Regulation of mRNA transcriptome by PI3K-AKT and MEK-ERK1/2 pathways

**Figure 2:**
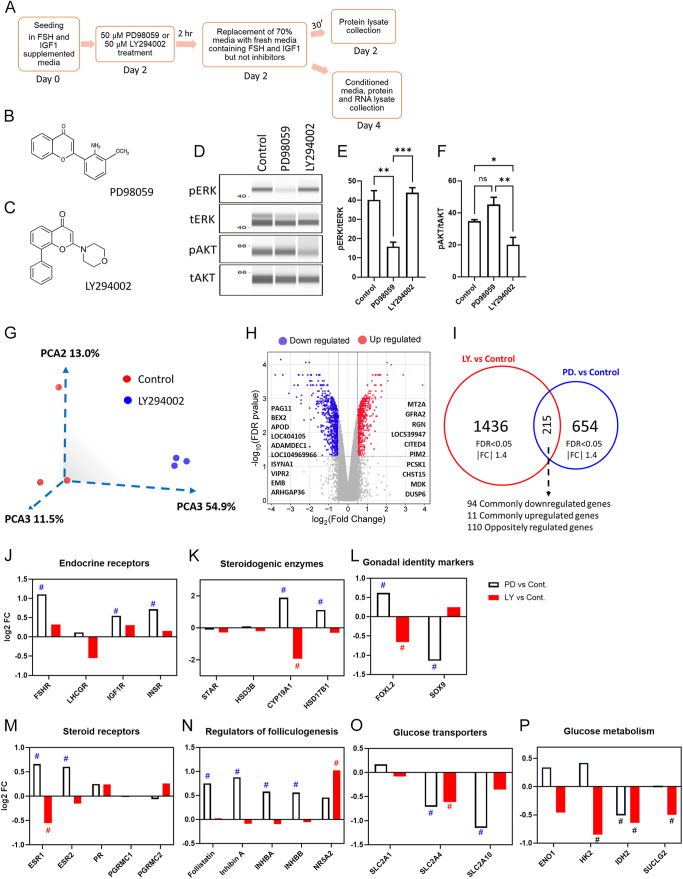
Comparative transcriptome changes induced by AKT and ERK pathways. (A) Schematic representation of cell culture experiments with inhibitor treatments. (B) and (C) Molecular structures of PD98059 and LY294002. (D) Representative western analysis images of AKT and ERK proteins in cells treated with PD98059 and LY294002. (E) and (F) Quantification of western analysis data, presented as mean ± SEM (*n* = 4) and analyzed using one-way ANOVA with Tukey’s post hoc test. (G) PCA clustering of vehicle control and LY294002-treated samples of the transcriptome analysis. (H) Volcano plot of transcriptome data generated by plotting log2 fold change values against −log10 FDR values in LY294002-treated cells. The top upregulated and downregulated genes in LY294002 are mentioned in the picture. (I) Venn diagram of differentially expressed genes between the AKT inhibitor-treated cells (LY vs control) and ERK inhibitor-treated cells (PD vs control). (J), (K), (L), (M), (N), (O) and (P) Expression of different genes encoding endocrine receptors (J), steroidogenic enzymes (K), gonadal markers (L), steroid receptors (M), regulators of ovarian folliculogenesis (N), glucose transporters (O) and glucose metabolism (P). These gene expression values were derived from the respective transcriptome datasets. The symbol # was given to the differentially regulated genes based on the criteria of FDR-adjusted *P* < 0.05 and fold change |1.4| from the microarray data. The *n* value indicates the number of independent cell culture experiments analyzed using granulosa cells collected on different days.

To determine the effect of PI3K-AKT signaling on global gene expression, we performed the microarray analysis of RNA isolated from granulosa cells treated with or without the signaling inhibitor LY294002. The schematic representation of cell culture and the chemical structure of inhibitors used in the experiments are shown in Fig. 2A, B, C. Treatment with PD98059 and LY294002 significantly reduced the levels of p-ERK and p-AKT, respectively, indicating the effectiveness of the inhibitors (Fig. 2D, E, F). Principal component analysis (PCA) indicated a distinct clustering of control and LY294002-treated samples (Fig. 2G). Expression of 1436 genes was found to be differentially regulated upon inhibiting PI3K-AKT signaling (volcano plot, Fig. 2H). Our earlier transcriptome analysis of granulosa cells treated with PD98059 identified that 654 genes were differentially regulated by the ERK pathway (Baddela *et al.* 2023). A comparison of differentially regulated genes in these two different studies revealed that 215 genes were found to be commonly regulated by the ERK and AKT pathways (Fig. 2I). Among them, 94 genes were downregulated (Supplementary Excel sheet 1 (see section on Supplementary materials given at the end of the article)) and 11 genes were upregulated (Supplementary Excel sheet 2) by both inhibitors. One hundred ten genes were found to be oppositely regulated by the AKT and ERK pathways (Supplementary Excel sheet 3). The remaining 1221 and 439 genes were uniquely regulated by the AKT and ERK pathways, respectively.

Several genes involved in endocrine signaling, steroidogenesis and transcriptional regulation in granulosa cells were regulated by the AKT and ERK signaling pathways. Notably, expression of FSH, IGF1 and insulin receptors was unaffected by inhibition of the PI3K-AKT pathway. In contrast, our previous study found these receptors were upregulated upon inhibition of the ERK pathway (Fig. 2J). Aromatase gene (*CYP19A1*) expression was downregulated by AKT inhibition (Fig. 2K), while it was induced by the ERK pathway in the previous analysis. We observed differential regulation of the key gonadal transcription factor *FOXL2 *by the AKT and ERK pathways (Fig. 2L). Expression of genes related to steroid receptors and genes involved in folliculogenesis is provided in Fig. 2M and N. Due to the partial hypoxic microenvironment in the ovarian follicles, granulosa cells depend greatly on glucose for energy metabolism. These transcriptome datasets showed that the expression of the glucose transporter 1 encoding *SLC2A1* was not regulated by these two pathways. In contrast, insulin-dependent *SLC2A4* expression was downregulated by inhibiting both AKT and ERK pathways (Fig. 2O). Furthermore, the rate-limiting glycolytic enzyme-encoding gene *HK2* (hexokinase 2), along with citric acid cycle genes *IDH2* (isocitrate dehydrogenase 2) and *SUCLG2* (succinyl-CoA ligase subunit beta), were downregulated by AKT inhibition (Fig. 2P). *IDH2* was also found to be downregulated upon inhibition of the ERK pathway (Baddela *et al.* 2023).

### Bioinformatic interpretations

**Figure 3:**
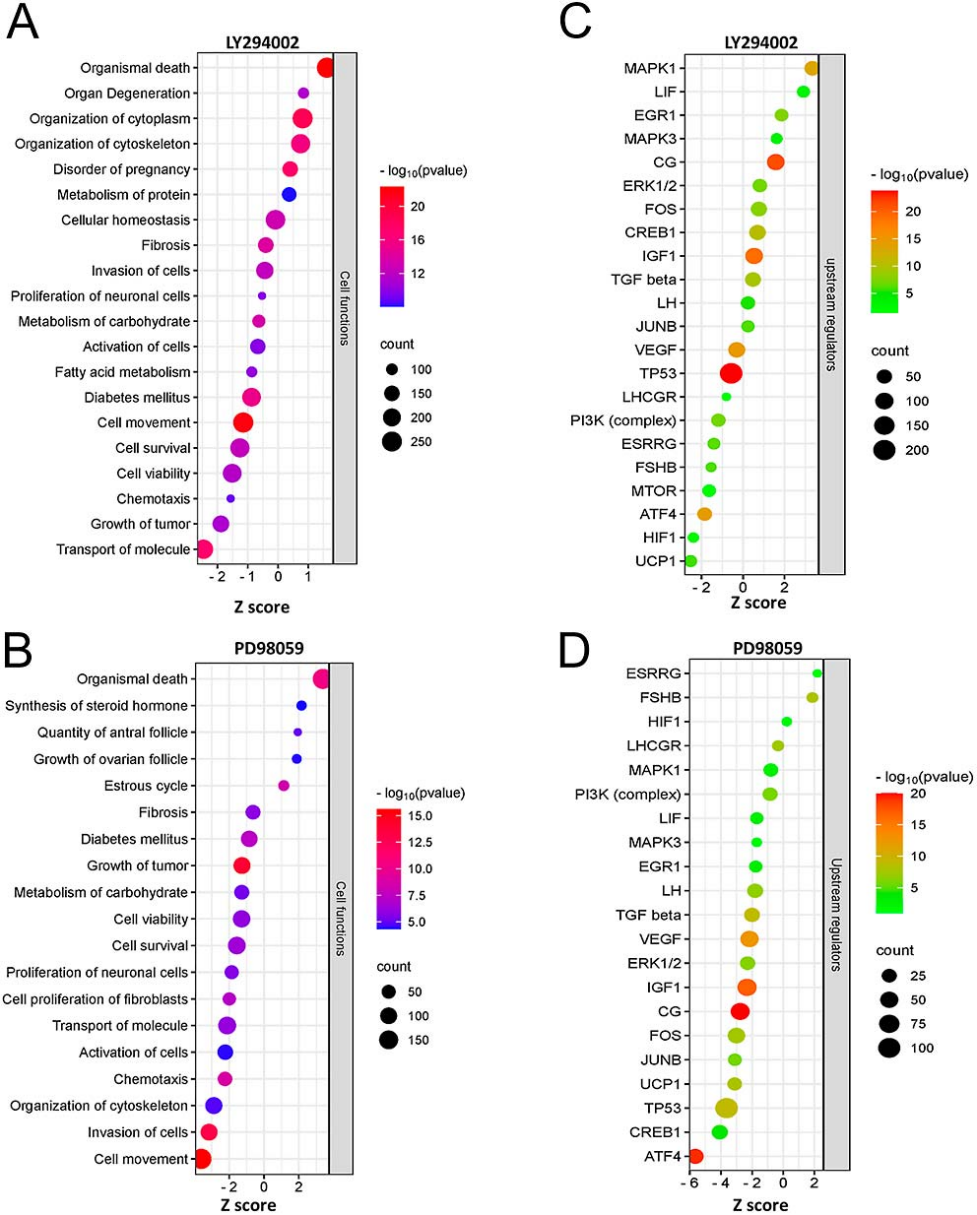
Cellular functions and upstream molecules regulated by AKT and ERK pathways in granulosa cells. (A) and (B) Significantly enriched cellular functions in LY294002- and PD98059-treated cells. (C) and (D) Significantly enriched upstream molecules in LY294002- and PD98059-treated cells. The *X*-axis indicates the activation score (*Z* score). The size of the circle indicates the gene count in the dataset corresponding to the particular cellular function or upstream molecule. The color of the circle indicates the −log10 *P* values.

The IPA showed that numerous cellular functions were impacted by the differentially regulated genes of the AKT and ERK signaling pathways (Fig. 3A and B; Supplementary Excel sheets 4 and 5). Vital cellular processes, such as molecule transport, growth, chemotaxis, cell viability and carbohydrate metabolism, were downregulated upon inhibiting either of these pathways. Interestingly, ovary-specific attributes, such as ‘steroid synthesis’, ‘estrous cycle’ and ‘growth of the ovarian follicle’ were found to be positively regulated upon inhibition of the ERK pathway, while ‘disorder of pregnancy’ was significantly enriched with a positive activation score upon inhibition of AKT signaling. Numerous upstream regulators were identified to be regulated by the AKT and ERK pathways (Fig. 3C and D; Supplementary Excel sheet 6 and Baddela *et al.* (2023)). Signaling by FSH, estrogen-related receptor (ESRRG) and hypoxia-inducible factor 1 (HIF1) were inhibited by the AKT inhibitor, whereas they were either increased or unaffected by the ERK inhibitor. Chorionic gonadotropin (CG), c-Fos (FOS), transforming growth factor beta (TGFβ), epidermal growth factor 1 (EGR1), LH and JUNB activities were inhibited by the ERK inhibitor and were either increased or unaffected by the AKT inhibitor. Regulators of energy metabolism, such as uncoupling protein 1 (UCP1) and activating transcription factor 4 (ATF4), were negatively regulated by both inhibitors.

### Regulation of steroidogenesis

**Figure 4:**
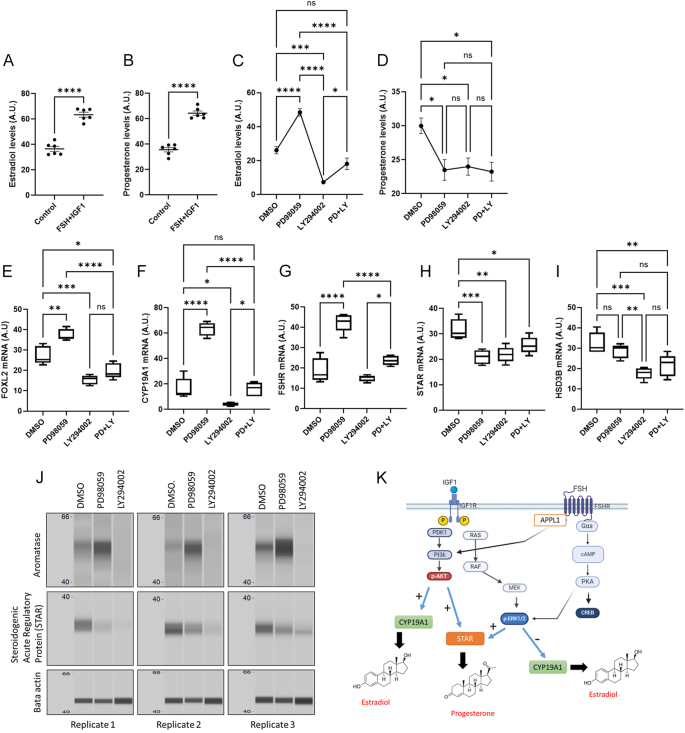
Regulation of steroidogenesis by AKT and ERK pathways. (A) and (B) Estradiol and progesterone levels in the culture media of cells treated with FSH and IGF1 compared to the untreated control (*n* = 6). (C) and (D) Estradiol and progesterone levels in the conditioned media of cells cultured with independent or combined treatment of inhibitors compared to DMSO control (*n* = 5). Data in (A) and (B) are presented as mean ± SEM and analyzed by unpaired *t*-test. Data in (C) and (D) are presented as mean ± SEM and analyzed by one-way ANOVA followed by Tukey’s post hoc test. (E), (F), (G), (H) and (I) Quantification of mRNA levels in qPCR for *FOXL2*, *FSHR*, *CYP19A1*, *STAR* and *HSD3B* genes (*n* = 5). Data in (E), (F), (G), (H) and (I) are presented as box plots and analyzed using one-way ANOVA followed by Tukey’s post hoc test. (J) Protein expression of aromatase, STAR and ACTB genes in FSH- and IGF1-treated cells incubated with DMSO, PD98059 and LY294002 in three different replicates. (K) Graphical representation of the regulation of estradiol and progesterone production by ERK and AKT pathways in granulosa cells under the stimulation of FSH and IGF1. This graphic was generated using bioRender.com. Probability values <0.05 were considered statistically significant and are designated with up to four asterisk symbols to inform the strength of significant difference (*P < 0.05; **P < 0.01; ***P < 0.001; ****P < 0.0001). ns, not significant. The *n* value indicates the number of independent cell culture experiments analyzed using granulosa cells collected on different days.

Transcriptome data indicated that inhibition of the AKT pathway downregulates the expression of *FOXL2* and *CYP19A1* genes involved in ovarian development and estradiol production, respectively. In contrast, inhibition of ERK induced the expression of these genes, suggesting that AKT induces and ERK inhibits estradiol production. To confirm these gene expression patterns coming from two different datasets, we measured estradiol levels in the culture media of granulosa cells. We also performed mRNA and protein analysis of cells under different treatments. As anticipated, FSH and IGF1 treatments induced estradiol and progesterone production compared to the control (Fig. 4A and B). The ERK inhibitor PD98059 significantly induced estradiol production, and the AKT inhibitor LY294002 decreased estradiol production (Fig. 4C). Combined treatment of inhibitors did not significantly impact estradiol levels compared to control but led to significant induction in comparison with LY294002 treatment. Both individual and combined treatments, however, reduced progesterone production (Fig. 4D). These results were further validated by qPCR analysis, which confirmed that PD98059 upregulated the expression of *FOXL2*, *CYP19A1* and *FSHR* genes, while LY294002 downregulated *FOXL2* and *CYP19A1* (Fig. 4E, F, G). Similar to estradiol levels, *CYP19A1* and *FSHR* expressions showed a slight but significant increase with PD+LY treatment compared to LY294002 alone. Both inhibitors also downregulated *STAR* expression, while *HSD3B* was downregulated by LY294002 and the PD+LY treatment (Fig. 4H and I). Furthermore, protein expression analysis also revealed that the aromatase levels encoded by the CYP19A1 gene were increased by the PD98059 treatment and downregulated in the LY294002 treatment (Fig. 4J). On the other hand, STAR protein levels are decreased by both inhibitors (Fig. 4J). Overall, these data indicate that AKT is involved in the induction of estradiol and progesterone production in granulosa cells, whereas ERK is involved in the inhibition of estradiol and induction of progesterone production (Fig. 4K).

### Glucose absorption and energy metabolism are disturbed by the AKT and ERK pathway inhibitors in varying degrees

**Figure 5:**
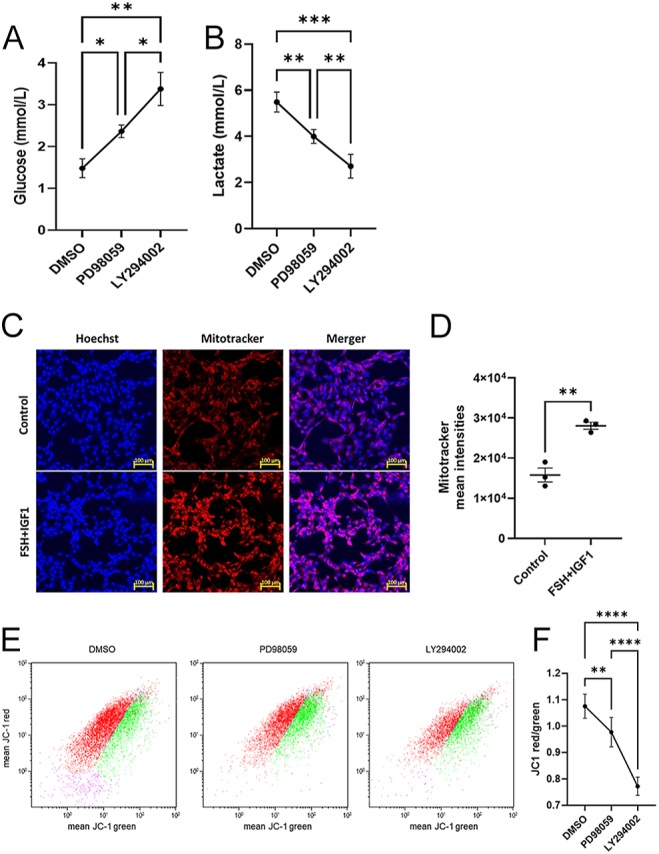
Regulation of glucose metabolism by AKT and ERK pathways. (A) and (B) Quantification of glucose and lactate levels in the spent culture media of granulosa cells (*n* = 4) treated with PD98059 and LY294002 compared to vehicle control (DMSO). Data are presented as mean ± SEM and analyzed by mixed model analysis followed by Tukey’s post hoc test. (C) Fluorescence microscopy images of granulosa cells stained with Hoechst and MitoTracker for analyzing the mitochondrial membrane potential (MMP). (D) MitoTracker quantification data presented as mean ± SEM of three independent cell cultures and analyzed by unpaired *t*-test. (E) Representative flow histograms of JC1 staining in vehicle control, PD98059- and LY294002-treated cells. The green dots indicate the JC1 monomers, and the red dots indicate the JC1 aggregates. (F) MMP (*n* = 6) quantification analyzed by RM one-way ANOVA followed by Tukey’s post hoc test. Probability values <0.05 were considered statistically significant and are designated with up to four asterisk symbols to inform the strength of significant difference (* *P* < 0.05; **P < 0.01; ***P < 0.001; ****P < 0.0001). The *n* value indicates the number of independent cell culture experiments performed using granulosa cells collected on different days.

Granulosa cells have been shown to be dependent on glycolysis for energy production, plausibly due to the partial hypoxic microenvironment of the follicle (Thompson *et al.* 2015, Baddela *et al.* 2020, Pan *et al.* 2022). Transcriptome data indicated that the inhibition of the AKT and ERK signaling pathways downregulated the expression of GLUT4 encoding *SLC2A4* gene. IPA identified that upstream regulators involved in glucose metabolism, including HIF1, ATF4 and UCP1 were being regulated by the AKT and ERK pathways. To ascertain this, we assayed glucose and lactate levels in the conditioned media. Results showed that the inhibition of the AKT and ERK pathways resulted in higher glucose levels in the culture media compared to control, with PI3K-AKT inhibition causing the strongest effect on glucose uptake (Fig. 5A). Lactate measurements showed exactly opposite measurements as the inhibition of the AKT and ERK pathways caused a significant decrease in lactate production, with AKT inhibition causing the lowest amount of lactate production (Fig. 5B). To analyze the metabolism down the lane to glycolysis, we have analyzed the MMP using either MitoTracker or JC-1 staining; FSH and IGF1 treatment significantly induced the MMP compared to the control as shown in the fluorescent microscopy images (Fig. 5C and D). Flow analysis revealed that treatment with PD98059 and LY294002 decreased MMP in the granulosa cells, with LY294002 causing the most substantial decrease in MMP (Fig. 5E and F), indicating that inhibition of these signaling pathways led to metabolic restriction in granulosa cells.

## Discussion

The dysregulation of the AKT and ERK pathways in granulosa cells could lead to impaired follicular development and infertility (Fan *et al.* 2009, Brown *et al.* 2010). The data presented in this article offer comprehensive genome-wide gene expression changes induced by the AKT and ERK signaling pathways, highlighting their distinct and overlapping aspects in regulating gene expression, steroidogenesis and metabolism in bovine granulosa cells. To our knowledge, this is the first report to discuss the comparative transcriptome changes induced by the PI3K-AKT and MEK-ERK pathways in any cell type. These data suggest that the activity of these pathways differs in regulating specific genes and corresponding cellular processes. The inhibition of PI3K-AKT signaling resulted in the differential expression of 1436 genes. This broad impact on the cellular transcriptome emphasizes a wide range of cellular processes and upstream molecules regulated by AKT signaling compared to ERK signaling, which affected 654 genes in granulosa cells. IPA revealed that genes regulated by both AKT and ERK pathways involved in fundamental cellular processes, such as chemotaxis, viability and proliferation, which are integral to increasing granulosa cell number and granulosa cell response to extracellular molecules during follicular development (Mebratu & Tesfaigzi 2009, Yu & Cui 2016).

Steroid production is a crucial aspect of granulosa cell function. During follicle development, pituitary FSH and hepatic IGF1 act on granulosa cells to induce estradiol production. The present analysis reveals that FSH-responsive genes were decreased in AKT inhibitor-treated cells. These genes were oppositely regulated in ERK inhibitor-treated cells. A clear distinction can be drawn between AKT and ERK pathways in terms of steroidogenesis as inhibition of PI3K-AKT signaling downregulated estradiol, whereas the inhibition of MEK-ERK signaling upregulated estradiol production via regulating *FOXL2* and* CYP19A1*. This observation is in line with earlier ERK1/2−/− mice model data, where the ovarian follicles were arrested in the preovulatory follicle stage with elevated estradiol but lower progesterone levels in the follicular fluid (Fan *et al.* 2009). However, other studies observed either no significant change or decreased aromatase gene expression in the cultured granulosa cells upon inhibiting ERK signaling (Mani *et al.* 2010, Huang *et al.* 2016). On the other hand, progesterone levels were decreased by both PD98059 and LY294002, suggesting that both these signaling pathways are critical for progesterone production. The transcriptome signals do not show significant regulation of *STAR* and *HSD3B* transcripts by the inhibitors. However, the qPCR validation revealed that LY294002 downregulates both genes, while PD98059 downregulates only *STAR*. Discrepancies in gene expression between microarray and qPCR data could be attributed to the binding specificity of the gene probes, as background signals and cross-hybridization of probes can reduce the sensitivity for some genes, which can cause pitfalls during signal quantification (Rachinger *et al.* 2021).

The transcriptome data revealed that the expression of *EGR1* and *JUNB* was increased by LY294002 and decreased by PD98059. Expression of these two genes was found to be increased in mouse granulosa cells treated with hCG for 1 h (Carletti & Christenson 2009). However, the expression of *EGR1* was also increased by FSH in rat granulosa cells via ERK signaling (Russell *et al.* 2003). Interestingly, EGR1-null mice were infertile due to impairment in ovulation and corpus luteum formation, similar to ERK1/2-null mice (Richards *et al.* 2002). Besides its role in ovulation, EGR1 also plays a role in the induction of ovarian fibrosis, which is observed in several conditions of subfertility. EGR1 induces the production of connective tissue growth factor CCN2, which accumulates in the ovarian stromal compartments and induces fibrosis (Han *et al.* 2020). Similarly, EGR1 was found to promote ovarian hyperstimulation syndrome through increasing SOX9 expression (Wang *et al.* 2023). Yuan *et al.* (2016) showed that EGR1 expression was significantly increased in aged mouse ovaries compared to the ovaries of young mice and increased follicular atresia in aged mice (Yuan *et al.* 2016). The increased expression of *EGR1* by LY294002 suggests that AKT signaling could also play a role in preventing ovarian fibrosis, ovarian hyperstimulation syndrome and follicular atresia via controlling the expression of *EGR1*.

The avascular nature of the granulosa and oocyte compartments in the ovarian follicles necessitates the activation of the HIF1 pathway for generating energy via glycolysis and efficient mitochondrial respiration. The present transcriptome suggested that HIF1 activity is inhibited upon blocking AKT signaling but not by ERK signaling. It was found that low levels of HIF1 expression in granulosa cell layers inhibit oocyte competence for fertilization in polycystic ovarian syndrome patients (Wang & Wu 2020). HIF1 activity was earlier found to be increased by FSH via activating the AKT signaling pathway. HIF1 induces the vascularization of the ovarian follicle during the dominant follicle development by upregulating the VEGF expression (Alam *et al.* 2004). A recent report by Liu and coworkers showed that HIF1 signaling protects the ovarian follicle from AMPK-induced atresia (Liu *et al.* 2023). Therefore, decreased glucose uptake in AKT inhibitor-treated cells could be partly mediated by the downregulation of the HIF1 signaling pathway. Activating transcription factor 4 (ATF4) is a member of the cAMP response element-binding (CREB) protein family. Transcriptome data suggest that ATF4 activity was decreased by the inhibition of both AKT and ERK pathways. ATF4 is involved in amino acid and glutathione metabolism, oxidative stress response and endoplasmic reticulum stress response (Lange *et al.* 2008). Interestingly, ATF4 was also proposed as a conserved regulator of cellular metabolism and carbohydrate homeostasis as ATF4-null mice are lean and resist diet-induced and age-induced obesity and diabetes (Seo *et al.* 2009). Under the present cell culture conditions, downregulation of ATF4 activity may denote reduced glucose availability due to its decreased uptake and metabolism in the inhibitor-treated cells.

Granulosa cells are known for their high glycolytic activity, producing pyruvate and lactate, which are essential for normal oocyte maturation (Biggers *et al.* 1967, Leese & Barton 1985, Kuchiiwa *et al.* 2011). Therefore, the inhibition of glucose uptake by PD98059 and LY294002 in the present study proportionately decreased lactate production and secretion into the media. Both AKT and ERK pathways are recognized for their roles in glucose metabolism (Yang *et al.* 2012, Li *et al.* 2024), and the current data suggest that AKT plays a more crucial role in energy metabolism than ERK in granulosa cells, as the most potent effects on glucose uptake and MMP were observed with LY294002 compared to PD98059 supplementation. ERK signaling appears to be involved in regulation rather than activation of steroidogenesis as cells treated with both PD98059 and LY294002 did not induce CYP19A1 gene expression compared to PD98059 alone.

Overall, the present transcriptome data offer a repository of genes regulated by the PI3K-AKT and MEK-ERK pathways in granulosa cells. Validation experiments confirm that the PI3K-AKT pathway functions as an activator, while the MEK-ERK pathway serves as a regulator of steroidogenesis. Both pathways are essential for energy metabolism, with AKT exerting a more pronounced effect than ERK. These data could inform future research about the novel genes regulated by the AKT and ERK pathways to determine their role in apoptosis, cell proliferation, metabolism, and other cellular processes in different cell systems.

## Supplementary materials



## Declaration of interest

The authors declare that there is no conflict of interest that could be perceived as prejudicing the impartiality of the work.

## Funding

This work is funded by Deutsche Forschungsgemeinschafthttps://doi.org/10.13039/501100001659, Bonn, Germany (DFG; grant No. BA 6909/1-1) to VSB.

## Author contribution statement

VSB conceived the research idea and designed the experiments. Experiments and analyses were performed by VSB, MM and XT. DK executed the microarray experiment at the University of Rostock, and JB helped in the IPA. VSB wrote the manuscript and analyzed the data. JV discussed the experiments, data and edited the manuscript.

## Data availability

The transcriptome data corresponding to PI3K-AKT signaling are deposited in Gene Expression Omnibus (GEO) following MIAME guidelines and can be accessed with the submission identification number GSE274651. The ERK signaling-regulated transcriptome data can be accessed with the identification number GSE225283. Website: https://www.ncbi.nlm.nih.gov/gds.
